# Gratitude and Adolescents’ Subjective Well-Being in School: The Multiple Mediating Roles of Basic Psychological Needs Satisfaction at School

**DOI:** 10.3389/fpsyg.2016.01409

**Published:** 2016-09-21

**Authors:** Lili Tian, Luyang Pi, E. S. Huebner, Minmin Du

**Affiliations:** ^1^Guangdong Key Laboratory of Mental Health and Cognitive Science, Center for Studies of Psychological Application, School of Psychology, South China Normal UniversityGuangzhou, China; ^2^Department of Psychology, University of South Carolina, ColumbiaSC, USA; ^3^Shajiao Junior High SchoolFoshan, China

**Keywords:** gratitude, basic psychological needs satisfaction at school, subjective well-being in school, multiple mediational role, adolescent

## Abstract

Based on the relation between gratitude and general subjective well-being (SWB), and Basic Psychological Needs Theory (Ryan and Deci, 2000), the present study’s aim was to use structural equation modeling to test the multiple mediational roles of the satisfaction of three basic psychological needs at school in accounting for the association between gratitude and SWB in school (school satisfaction, school affect) in adolescents. A total of 881 Chinese adolescents (427 males; Mean age = 12.97) completed a multi-measure questionnaire that tapped the targeted variables. Findings revealed that gratitude related significantly, positively to adolescents’ SWB in school. Moreover, a multiple-mediators analysis suggested that relatedness and competence needs satisfaction at school mediated the relation between gratitude and SWB in school. Lastly, a multiple-mediators analysis also indicated that autonomy needs satisfaction mediated the relation between relatedness and competence needs and SWB in school. Limitations and practical applications of the study were discussed.

## Introduction

With the development of positive psychology, gratitude has become a popular research topic of human functioning. A large body of research has shown that gratitude is positively associated with *general* subjective well-being (SWB) in adults (see Wood et al., 2010, for a review). However,in recent years, some researchers have begun to focus on the relations between gratitude and domain-specific SWB reports, such as sports (Chen and Kee, 2008) and family (Hoy et al., 2013). Such research is consistent with the notion of Sarason (1997) that “subjective well-being judgments always occur within environmental contexts, such as relationships, family and school” (p. 9). Nevertheless, scant attention has been paid to gratitude and SWB *in school* in particular (Long et al., 2012). The time and energy that students devote to schooling implies that school represents a major institutional context for students’ learning and social-emotional development. Students’ experiences in their schools significantly contribute to their lifelong development (Park, 2004). In particular, gratitude experiences appear crucial in school life in that frequent experiences of gratitude have been significantly associated with students’ academic, psychological and social well-being (see Froh and Bono, 2011, for a review). Given that adolescence is an important stage of cognitive, emotional, and social maturation (Steinberg, 2005) and that higher levels of SWB in school are also closely related to adolescents’ learning and social outcomes (Suldo et al., 2014), it seems important to understand the development and consequences of individuals’ SWB in school. Therefore, the present study focused on elucidating further the relations between gratitude and SWB in school in adolescents in particular.

Basic psychological needs theory (BPNT; Ryan and Deci, 2000) proposes that humans have three basic psychological needs: relatedness, competence, and autonomy. Previous research (e.g., Milyavskaya et al., 2009; Eryilmaz, 2012) has shown that the satisfaction of the three basic psychological needs promotes individuals’ overall SWB. Tian et al. (2016) also found that basic psychological needs satisfaction at school had a positive effect on students’ SWB in school. Although the association between basic needs satisfaction and adolescents’ SWB in school was significant, we know of no study that has examined the association between SWB and gratitude and the possible mediating role of the satisfaction of the three basic psychological needs at school simultaneously. On the other hand, scant attention has been paid to the relations among gratitude, basic psychological needs, and SWB in the context of the specific domain of school (Long et al., 2012; Tian et al., 2016). In addition, specification of the particular psychosocial mechanisms that explain the association between gratitude and SWB in school (e.g., through the satisfaction of the three basic psychological needs at school) is crucial to elucidate the basis for the development of empirically based interventions to enhance adolescents’ SWB in school. Thus, this study specifically examined (1) the link between gratitude and SWB in school in adolescents and (2) the mediating roles of the satisfaction of the three basic psychological needs at school in accounting for this relation between gratitude and SWB in school.

### Subjective Well-Being in School

Diener’s (1994) conceptual framework for SWB is widely used. According to Diener’s theory, separable cognitive (i.e., the satisfaction with life) and affective components (i.e., positive and negative feelings) comprise an individual’s overall SWB (Diener et al., 1999). Based on Diener’s theory, Tian (2008) offered a theoretical model of children’s SWB in school, which incorporates students’ cognitive and affective well-being, specifically as experienced in the school context. SWB in school in Tian’s (2008) model is composed of students’ reports on school satisfaction (SS) and affect in school (AS). As part of their SWB, students report on their SS, which is a student’s cognitive evaluation of school life in general. In doing so, students evaluate several domains of school life based on their own standards, such as their satisfaction with their academic studies and satisfaction with relationships between students and teachers. Students also report on the positive and negative feelings that they experience during school. Grounded in this model, the Brief Adolescent’s Subjective Well-Being in School Scale (BASWBSS) was proposed by Tian et al. (2015b), in which has displayed good psychometric properties in adolescents in China.

### Gratitude and SWB in School

Gratitude can be defined as a sense of real-time emotional experience, such as thankfulness and joy in response to receiving a gift (McCullough et al., 2002). Research has shown that gratitude is one of the most important factors that influences individuals’ overall happiness (Froh and Bono, 2008) and is thus an important basis for experiencing happiness in individuals (Watkins et al., 2003). For example, Watkins et al. (2003) found that gratitude may help individuals’ experience more positive emotions, happiness, and hope in their lives in general. Froh et al. (2009) also found that adolescents who reported grateful moods indicated greater SWB. Extending beyond such studies, however, researchers have recently begun to focus attention on the experience of gratitude using a more contextualized approach, such as studies of gratitude within specific life domains (e.g., school). Many studies have shown that frequent experiences of gratitude are conducive to the healthy development of students. For instance, Wood et al. (2010) found that students reporting higher levels of gratitude are more likely to appreciate their teachers and peers in school. Wen et al. (2010) demonstrated that junior high school students who report higher levels of gratitude also report higher academic achievement, and higher academic achievement often yields happiness (Ng et al., 2015). Thus, we hypothesized that gratitude will be related significantly, positively to SWB in school in adolescents.

### Basic Psychological Needs Satisfaction at School as a Mediator

Based on BPNT (Ryan and Deci, 2000), Tian et al. (2014) extended BPNT to the domain of adolescents’ school life. She put forward that autonomy, relatedness, and competence were three basic psychological needs for adolescent students at school. Autonomy needs at school represent students’ experiences of strength of will and self-endorsement of their school behavior. Relatedness needs at school represent students’ feelings of belonging in school, which includes their connections with their teachers and classmates. Competence needs at school represent students’ experiences related to the development and expression of their personal abilities in school. Tian et al. (2014) also developed a scale for evaluating adolescent students’ basic psychological needs at school (Adolescent Students’ Basic Psychological Needs at School Scale, ASBPNSS).

Numerous studies support the notion that needs satisfaction is linked to general or overall SWB in adolescents (e.g., Véronneau et al., 2005; Leversen et al., 2012). Nevertheless, most of these research studies examined overall SWB, rather than domain-specific SWB, such as SWB in school. Recently, a few studies have addressed the associations between needs satisfaction and SWB in school in adolescents (Tian et al., 2016). For instance, Tian et al. (2016) found that basic psychological needs satisfaction at school has a positive effect on SWB in school. However, studies of basic psychological needs satisfaction at school and SWB in school remain sparse, leaving much to be done to clarify our understanding of the relational mechanism between psychological need satisfaction and domain-specific SWB.

Researchers have also demonstrated that higher levels of gratitude exert a positive effect on basic psychological needs satisfaction. For example, Wood et al. (2009) found that gratitude is related significantly to college students’ autonomy, relatedness, and competence needs satisfaction after controlling for the Big Five personality traits. Kashdan et al. (2009) also observed significant, positive relations between gratitude and satisfaction of autonomy, relatedness, and competence needs in adults. Consistent with Fredrickson’s (2004) broaden and build theory of positive emotions, frequent experiences of the positive emotion of gratitude should lead to expanded cognitive and social resources, which should help individuals satisfy their competence and relatedness needs. Although theory and empirical research suggest that gratitude can promote basic psychological needs satisfaction in general, researchers have not demonstrated that gratitude is related to the satisfaction of domain-specific needs satisfaction; for example, satisfaction of fundamental needs in the school environment in particular. It is also important to note that previous research has generally treated the three components of basic psychological needs as an aggregate (i.e., combining reports across all three needs) or “parallel” variables, ignoring the interconnections among the three components. Yu et al. (2012) argued it is inner strength (competence) and external support (relatedness), which allow individuals to influence their environments and make their own decisions (autonomy). Based on the application of this notion to the experience of basic psychological needs satisfaction at school, we proposed the following corollary: Students who experience feelings of competence and relatedness (with teachers and other students) in school will experience a greater sense of autonomy in school. Therefore, this study assumes not only that psychological needs satisfaction at school should mediate the relation between gratitude and SWB in school; but also that gratitude should relate to the satisfaction of competence and relatedness needs at school, which in turn should relate to the satisfaction of autonomy needs at school, which in turn should relate to adolescents’ SWB in school.

## The Current Study

This study added to the literature on SWB in school through examining the influence of gratitude on adolescents’ SWB in school through the satisfaction of the three basic psychological needs at school. Based on the aforementioned literature and BPNT, the present study tested the relations among gratitude, satisfaction of three basic psychological needs at school, and SWB in school via structural equation modeling (SEM). Specifically, Three hypotheses were formulated: (1) gratitude will relate significantly to SWB in school; (2) psychological needs satisfaction at school will mediate the relation between gratitude and SWB in school; (3) gratitude will relate to the satisfaction of competence need at school, which in turn will relate to the satisfaction of autonomy needs at school, which in turn will relate to the SWB in school. Furthermore, gratitude will relate to the satisfaction of relatedness needs at school, which in turn will relate to the satisfaction of autonomy needs at school, which in turn will relate to SWB in school.

## Materials and Methods

### Participants

This study employed convenience samples from public schools located in northern China. These schools were typical, coeducational schools; they were all fairly representative of schools in that province, based on the information given by the local education entities. The schools were comparable as to school size, class size, teachers’ teaching ability, and as quality of students. A total of 910 students (445 males and 465 females) participated in the survey. Eleven females and 18 males did not complete the survey. These students were excluded from the analysis because of the missing data in their surveys. Thus, the quantity of valid respondents was 454 females and 427 males, yielding response rates of 97.63 and 95.96%, individually. The average age of these respondents was 12.97 years, ranging in age from 11 to 15 years (*SD* = 0.67). Virtually all respondents came from middle-income families in which parents at least had a high school degree.

### Measures

#### Gratitude

The Gratitude Questionnaire (GQ-6; McCullough et al., 2002) was used to measure gratitude. It includes six self-report items, such as “I am grateful to a wide variety of people”. A 7-point Likert scale ranging from 1 (strongly disagree) to 7 (strongly agree) is typically used for participants’ responses. However, based on prior research, Chinese individuals show a tendency to be noncommittal in their self reports (Chen and Kee, 2008). To reduce the likelihood of noncommittal responses, we thus deleted the neutral response option, reducing the 7-point Likert scale to a 6-point Likert scale. Mean scores were calculated, and higher scores reflected higher levels of gratitude. Evidence has been provided to support the validity of GQ-6 for use with Chinese school students (Tian et al., 2015a). In the present study, the Cronbach’s alpha coefficient was 0.76.

#### Subjective Well-Being in School

Subjective well-being in school was measured using the BASWBSS (Tian et al., 2015b). The BASWBSS is an 8-item self-report scale comprised of two subscales: SS and AS. The SS subscale consists of six items (e.g., “The teachers’ instructional methods and quality are good.”). Items were rated on a 6-point scale, with response options ranging from 1 (strongly disagree) to 6 (strongly agree). The AS subscale consists of two items. One item assessed the frequency of positive affect (PA) in school, and the other item assessed the frequency of negative affect (NA) in school. The PA item was worded as “In school, the frequency of my pleasant feelings is…”. The NA item was worded as “In school, the frequency of my unpleasant feelings is…”. Participants responded to both items using a 6-point response option scale, ranging from 1 (never) to 6 (always). The SS subscale score was calculated through averaging the responses to the six items. The AS subscale score was calculated through subtracting the NA from the PA score. Ultimately, the SS and AS subscale scores were summed to generate a total BASWBSS score. In Chinese adolescents, the SWB in school model and the BASWBSS has garnered empirical support (Tian et al., 2015b). In the present study, the Cronbach’s alpha coefficient for the SS subscale was 0.77.

#### Autonomy Needs Satisfaction at School

Autonomy needs satisfaction at school was measured by Need for Autonomy subscale of Adolescent Students’ Basic Psychological Needs at School Scale (ASBPNSS; Tian et al., 2014), which consists of 5 items (e.g., “I can decide for myself how to do things at school”). Participants responded on a 6-point Likert scale, ranging from 1 (strongly disagree) to 6 (strongly agree). Mean scores were calculated, and higher scores reflected higher levels of autonomy needs satisfaction at school. Tian et al. (2016) reported an internal consistency coefficient of 0.84. The Cronbach’s alpha coefficient in the present study was 0.86.

#### Relatedness Needs Satisfaction at School

Relatedness needs satisfaction at school was measured by the Need for Relatedness subscale of the ASBPNSS, which consists of five items (e.g., “Teachers and classmates care about me at school”). Participants responded on a 6-point Likert scale, ranging from 1 (strongly disagree) to 6 (strongly agree). Mean scores were calculated. Higher scores represented higher levels of relatedness needs satisfaction at school. Tian et al. (2016) reported an internal consistency of 0.79. The Cronbach’s alpha coefficient in the present study was 0.76.

#### Competence Needs Satisfaction at School

Competence needs satisfaction at school was measured by the Need for Competence subscale of the ASBPNSS, which consists of five items (e.g., “I am capable of learning new knowledge at school.”). Participants responded on a 6-point Likert scale, ranging from 1 (strongly disagree) to 6 (strongly agree). Mean scores were calculated. Higher scores represented higher levels of competence needs satisfaction at school. The internal consistency recorded in the study Tian et al. (2016) was 0.69. The Cronbach’s alpha coefficient obtained in the present study was 0.74.

### Procedure

This study received approval from the Human Research Ethics Committee of South China Normal University and from the related school boards, principals, as well as teachers. Besides these approvals, letters explaining this study and consent forms were delivered to the participants’ parents. Only the participants whose parent signed and returned the consent forms and who sent their own assent participated in this study. The measures were administered to groups of about 50 students by a trained graduate assistant in a classroom in their school. Identical written and verbal instructions were given to the participants by the trained assistant. The participants were given as much time as required to finish the questionnaire. After the completion of all measures, these students were debriefed about the objective of this research. Beyond the standard measures mentioned previously, students were also required to offer information about gender and age.

### Data Analysis

Given our relatively large sample size and small amount of missing data (i.e., 3.19%), the missing data were handled using the listwise deletion procedure, which is acceptable when the loss of cases due to missing data is less than 5% (Graham, 2009). Data analyses subsequently included three procedures. First, SPSS 17.0 was employed to obtain the descriptive data and Pearson correlations. Second, the two-step process developed by Anderson and Gerbing (1988) was adopted to analyze mediation effects. Specifically, the measurement model was evaluated to determine the extent to which its indicators reflected each of the latent variables. After evaluating the measurement model, the maximum likelihood value was used to evaluate the structural model and the value was chosen from AMOS 17.0. Multiple indicators were used to evaluate model fit, including the Tucker Lewis index (TLI), the Incremental Fit index (IFI), the comparative fit index (CFI), and the root mean-square error of approximation (RMSEA) (Anderson and Gerbing, 1988; Hoyle, 1995). For the CFI, IFI, and TLI indices, values over 0.90 are generally consider acceptable, while values over 0.95 are seen as a good fit. As for the RMSEA, values lower than 0.06 are generally regarded as a good fit, while values from 0.06 to 0.10 are regarded as acceptable (Hu and Bentler, 1999; Byrne, 2001). Finally, bootstrapping, employing 1000 samples, was used for testing the significance of the mediated effects and to produce bias-corrected percentile confidence intervals (MacKinnon, 2008). The size of the mediating effect was also calculated.

## Results

### Descriptive Analyses

Results in **Table 1** indicated that our data were normally distributed. Univariate skewness higher than or equal to 2.0, and kurtosis higher than or equal to 7.0 is regarded as moderate to high non-normality; problems in analyses may emerge with such levels of non-normality (Muthén and Kaplan, 1992; Curran et al., 1996). Nevertheless, all the variables in this study showed much lower values.

**Table 1:** Descriptive statistics and correlations for the observed variables (*N* = 881).

Variable	*M*	*SD*	Skew	Kurt	1	2	3	4
Gratitude	4.89	0.85	-0.81	0.68	–			
Autonomy	3.89	1.16	-0.43	-0.26	0.17^∗∗^	–		
Relatedness	4.98	0.85	-0.82	0.19	0.31^∗∗^	0.34^∗∗^	–	
Competence	4.32	0.92	-0.46	-0.09	0.34^∗∗^	0.33^∗∗^	0.49^∗∗^	–
SWBS	6.53	2.24	-0.63	0.55	0.27^∗∗^	0.41^∗∗^	0.68^∗∗^	0.48^∗∗^


*Autonomy = the satisfaction of autonomy needs at school; Relatedness = the satisfaction of relatedness needs at school; Competence = the satisfaction of competence needs at school; SWBS = subjective well-being in school. ^∗∗^*p* < 0.01.*

Analyses of all study variables, presented in **Table 1**, showed that the correlations among gratitude, the satisfaction of autonomy needs at school, the satisfaction of relatedness needs at school, the satisfaction of competence needs at school and SWB in school were all positive and significant at the *p* < 0.01 level. Of the three psychological needs, satisfaction of relatedness need at school had the strongest correlation with SWB in school (*r* = 0.68).

### The Measurement and Structural Models

**FIGURE 1:**
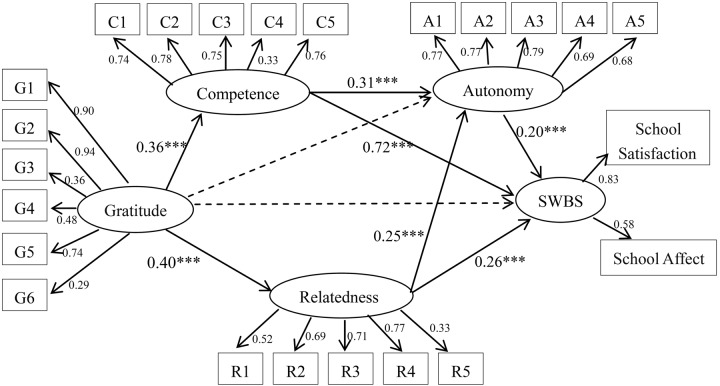
**The Mediation Model (*N* = 881).** G1–G6 = six items of gratitude; C1-C5 = five items of competence; A1-A5 = five items of autonomy; R1-R5 = five items of relatedness; Autonomy = the satisfaction of autonomy needs at school; Relatedness = the satisfaction of relatedness needs at school; Competence = the satisfaction of competence needs at school; SWBS = subjective well-being in school. ^∗∗∗^*p* < 0.001.

The measurement model consisted of five latent factors (gratitude, the satisfaction of autonomy needs at school, the satisfaction of competence needs at school, the satisfaction of autonomy needs at school, and SWB in school) and 23 observed variables. An initial examination of the measurement model showed an acceptable fit to the data: CFI = 0.95, TLI = 0.94, IFI = 0.95, RMSEA = 0.05. The latent variables’ factor compositions are portrayed in **Figure 1**. The measured variables’ loadings on the latent variables were all statistically significant (*p* < 0.001), which indicated that the latent variables were adequately measured by their indicators.

The direct path coefficients from predictor (gratitude) to the criterion (SWB in school, β = 0.35, *p* < 0.001) were meaningful. A model considering all the paths from gratitude to SWB in school revealed an acceptable fit to the data, CFI = 0.95, TLI = 0.94, IFI = 0.95, RMSEA = 0.05. However, the standardized path coefficient from gratitude to SWB in school became non-significant (β = -0.01, *p* > 0.05). Additionally, the standardized path coefficient from gratitude to autonomy satisfaction was also non-significant (β = -0.01, *p* > 0.05). Therefore, we constrained the two paths to zero to determine whether doing so worsened the fit of the model to the data. Nevertheless, the model fit remained acceptable: CFI = 0.95; TLI = 0.94; IFI = 0.95; RMSEA = 0.05. Although the difference in fit between the models was small, the greater parsimony of the modified model suggested that its fit was more satisfactory.

### Mediating Analysis

The statistical significance of the indirect effects in the mediation model was examined by using bootstrap process suggested by Shrout and Bolger (2002). By random sampling with replacement, a number of samples such as 1,000 are obtained from the initial data in the procedure of bootstrapping. In each of these bootstrap samples, the indirect effect is then calculated. A total of 1000 bootstrapping samples were generated by us from the original data set (*N* = 881) through random sampling. We drew the conclusion that the indirect effect was statistically meaningful at the level of 0.05 if the 95 % confidence interval for the indirect effect estimate did not contain zero (see Shrout and Bolger, 2002). In **Table 2**, the results show that the 95% CI for the four indirect effects did not contain zero, which indicates that all of the indirect effects were statistically significant. The mediating effect of relatedness satisfaction on SWB in school was 0.26, explaining 63.4% of the overall effects (0.41). The mediating effect of competence satisfaction on SWB in school was 0.11, explaining 26.8% of the overall effects. Through relatedness satisfaction and autonomy satisfaction, the mediating effect on SWB in school was 0.02, which explained 4.9% of the overall effects. Through competence satisfaction and autonomy satisfaction, the mediating effect on SWB in school was 0.02, which explained 4.9% of the overall effects.

**Table 2:** Bootstrap analyses of the magnitude and statistical significance of indirect effects.

Model pathways	β standardized indirect effect	B mean indirect effect^a^	SE of mean^a^	95% CI mean indirect effect^a^(lower and upper)
Gratitude-Relatedness-SWBS	0.36^∗^0.72 = 0.26	0.389	0.0047	0.241, 0.626
Gratitude-Competence-SWBS	0.40^∗^0.27 = 0.11	0.160	0.0060	0.068, 0.324
Gratitude-Relatedness-Autonomy-SWBS	0.36^∗^0.30^∗^0.20 = 0.02	0.032	0.0002	0.009, 0.083
Gratitude-Competence-Autonomy-SWBS	0.40^∗^0.26^∗^0.20 = 0.02	0.030	0.0008	0.006, 0.081


*Autonomy = the satisfaction of autonomy needs at school; Relatedness = the satisfaction of relatedness needs at school; Competence = the satisfaction of competence needs at school; SWBS = subjective well-being in school. ^a^These values are based on unstandardized path coefficients.*

## Discussion

### Relations between Gratitude and SWB in School

We evaluated the direct relation between gratitude and adolescents’ SWB in school. As expected, the result showed that gratitude demonstrated a significant, positive relation with adolescents’ SWB in school. This finding was consistent with previous studies in adults (Emmons and McCullough, 2003; Froh et al., 2008; Tian et al., 2015a). Previous research with adolescents has revealed significant relations with important variables, including higher levels of achievement motivation (Bono and Froh, 2009) and lower levels of burnout in learning (Froh et al., 2011). Using an experimental design, Froh et al. (2008) demonstrated that adolescents who completed daily gratitude diaries showed increases in optimism, positive emotions, and satisfactions. Thus, the research to date supports continued consideration of the importance of gratitude in students’ experiences in school.

### Multiple Mediational Role of Basic Psychological Needs Satisfaction at School

We also explored possible psychosocial mediators of the observed link between gratitude and SWB in school. Specifically, we investigated whether basic psychological needs satisfaction mediated the relation between gratitude and SWB in school. Using SEM, the results suggested multiple mediators. That is, our SEM results showed that both the satisfaction of relatedness needs and the satisfaction of competence needs at school served as significant mediators of the relation between gratitude and SWB in school, which partially supported our second hypothesis. Thus, gratitude not only can directly relate to adolescents’ SWB in school, but it also can indirectly relate to their SWB in school by satisfying adolescents’ relatedness needs at school and competence needs at school. Such results are consistent with notions of the relation of gratitude and achievement motivation (see Bono and Froh, 2009), which posit that gratitude inspires individuals’ goals and pursuits, promoting participation in schools and society, and thus encouraging individuals to obtain greater academic success and a richer social network. Whereas gratitude and achievement motivation theory and research have both traditionally been contextualized within the broad scope of individuals’ *overall* lives, our study is situated within the specific context of school life, thus addressing their generalizability to the specific domain of adolescents’ lives in school.

Related to hypothesis 3, we found that adolescents’ gratitude related to the satisfaction of their relatedness needs and competence needs at school, which in turn related to the satisfaction of their autonomy needs at school, and which in turn related to their SWB in school. Gratitude appears to help students experience a sense of connection with classmates and teachers and a feeling of school belonging, which is conducive to satisfy their relatedness needs during school. Gratitude also appears to relate to more successful experiences in students’ school lives, which supports the satisfaction of competence needs in the school context. It seems to follow that the satisfaction of relatedness and competence needs in school in particular, in turn allows students to experience strength of will and self-endorsement of their school behavior, which is conducive to satisfying autonomy needs and enhancing their SWB in school. The findings are consistent with Danielsen et al. (2009) who found that the satisfaction of relatedness needs not only strengthened the bonding between students, but also that positive student-to-student interactions may contribute to the fulfillment of students’ needs for autonomy through a shared focus on learning activities. In addition, our findings are also consistent with those of Kashdan et al. (2009) with adults, in which grateful individuals appeared to be more aware of and accepting of positive social connections, which in turn provided them with greater confidence to act autonomously and express individual, core values.

Finally, our study also revealed that gratitude did not directly relate to the satisfaction of adolescents’ autonomy needs at school, suggesting that gratitude indirectly relates to autonomy needs satisfaction through the satisfaction of relatedness and competence needs at school. This finding is also consistent with the study of Kashdan et al. (2009) who demonstrated that relatedness and competence needs satisfaction can promote autonomy need satisfaction. This finding may explain why our second hypothesis was partially supported.

### Limitations and Future Direction

The present study had several noteworthy limitations. First, cross-sectional data impose restrictions on claims of causality. In our models, we assumed that gratitude leads to greater SWB in school through the satisfaction of three basic psychological needs at school. Although the model for this study is based on cross- sectional data, a large number of studies of gratitude and general SWB previously supported the proposed direction of causality, specifically Gratitude → SWB (see Wood et al., 2010, for a review). Furthermore, previous studies based on experimental (Emmons and McCullough, 2003) and longitudinal research methodology (Lyubomirsky et al., 2011) have also supported this particular specification of directionality; thus making this the dominant view of the relation between these variables. Therefore, we made the same assumption in our model of the origins of SWB in school. Meanwhile, longitudinal research (Sheldon and Elliot, 1999) also has supported the notion that needs satisfaction leads to greater SWB. However, it remains possible that greater SWB in school increases gratitude through the satisfaction of three basic psychological needs at school. Future, more stringent work with longitudinal designs is necessary to pinpoint the causal directions among the variables. Second, all data were derived from self-reports of students. Multi-method assessments would build confidence in the validity of the findings, although the validity of adolescents’ self-report measures has been demonstrated in prior research (e.g., Dew and Huebner, 1994; Gilligan and Huebner, 2002). Third, we focused on the relations between gratitude and SWB in school in Chinese adolescents; further cross-age and inter-cultural research is needed to evaluate the universality of our findings (Emmons and Shelton, 2002). Finally, we addressed basic psychological needs satisfaction at school as a mediating variable. Other mediators, such as self-esteem, should be tested in further investigations to understand the full array of possible mediating mechanisms.

### Implications

This study demonstrates that Chinese adolescents’ gratitude significantly and positively related to their SWB in school. This result suggests that educators and parents may wish to consider employing empirically validated methods to enhance students’ SWB in school by the cultivation of students’ gratitude in suitable circumstances. For example, students could be requested to participate in gratitude interventions like “counting blessings”, in which they are instructed to complete gratitude diaries, listing up to six things they feel grateful for, daily for 2 weeks (Geraghty et al., 2010). Additionally, we found that, in school life, gratitude can indirectly relate to adolescents’ SWB in school through the psychosocial mechanisms of satisfaction of competence needs and relatedness needs at school. Therefore, school professionals may also wish to consider systematic efforts to improve students’ SWB in school by satisfying their competence and relatedness needs (Doll et al., 2014). Finally, the path Gratitude → competence needs and relatedness needs satisfaction at school → autonomy needs satisfaction at school → SWB in school illuminates the differentially important roles of basic psychological needs in students’ psychological functioning. These findings not only extend BPNT to the field of education, but they should contribute to the growing body of research that provides tentative implications for specific needs-based interventions aimed at promoting experiences that are conducive to positive SWB in school among adolescents (Suldo et al., 2014).

## Author Contributions

All the authors (LT, LP, EH, and MD) substantially contributed to the conception and the design of the work. LT, LP, and MD participated to the acquisition of data. The two first authors (LT and LP) analyzed and interpreted the data. The first author (LT) prepared the draft and the contributing authors (LP and EH) reviewed it critically and gave important intellectual content. All the authors (LT, LP, EH, and MD) worked for the final approval of the version to be published. All the authors (LT, LP, EH, and MD) are accountable for all the aspects of the work in ensuring that questions related to the accuracy or integrity of any part of the work are appropriately investigated and resolved.

## Conflict of Interest Statement

The authors declare that the research was conducted in the absence of any commercial or financial relationships that could be construed as a potential conflict of interest.
